# Vitamin C Enhances the Antibacterial Activity of Honey against Planktonic and Biofilm-Embedded Bacteria

**DOI:** 10.3390/molecules25040992

**Published:** 2020-02-23

**Authors:** Juraj Majtan, Martin Sojka, Helena Palenikova, Marcela Bucekova, Viktor Majtan

**Affiliations:** 1 Laboratory of Apidology and Apitherapy, Department of Microbial Genetics, Institute of Molecular Biology, Slovak Academy of Sciences, Dubravska cesta 21, 845 51 Bratislava, Slovakia; palenikova.helenaa@gmail.com (H.P.); marcela.bucekova@gmail.com (M.B.); 2 Department of Microbiology, Faculty of Medicine, Slovak Medical University, Limbova 12, 833 03 Bratislava, Slovakia; martin.sojka1@gmail.com (M.S.); viktor.majtan@szu.sk (V.M.); 3 Regional Health Public Authority, Medercska 39, 945 75 Komarno, Slovakia; 4 Protein Science Lab, Department of Biological Sciences, University of Singapore, Singapore 119077, Singapore

**Keywords:** antibacterial activity, hydrogen peroxide, biofilm, wound

## Abstract

Multifactorial antibacterial action is an important feature of honey; however, its bactericidal efficacy against biofilm-embedded bacteria is limited. The aim of this study was to investigate the impact of vitamin C (Vit C) on the antibacterial activity of natural honeys against planktonic as well as biofilm-embedded bacterial pathogens. The antibacterial activity of four honey samples supplemented with Vit C was expressed as the minimum inhibitory concentration (MIC). At sub-MICs, Vit C significantly increased the antibacterial activity of the tested honeys against *Pseudomonas aeruginosa* in planktonic cultures. However, after supplementation, honeydew honey, the most active honey, was ineffective against *Staphylococcus aureus*. On the other hand, when 100% honeydew honey was supplemented with Vit C (100 mg/g of honey) in a multispecies wound biofilm model, complete eradication of almost all bacterial isolates, including *S. aureus*, was observed. Furthermore, a mixture of honey and Vit C was partially effective against *Enterococcus faecalis*, whereas honey alone exhibited no antibacterial activity against this bacterium. Vit C counteracted hydrogen peroxide in honey solution and, thus, eliminated the major antibacterial compound present in honey. It is likely that a combination of honey with Vit C may trigger the intracellular production of reactive oxygen species in bacterial cells, but the exact cellular mechanisms warrant further investigations.

## 1. Introduction

Honey has been successfully used as a topical wound care product, with pronounced antibacterial, antibiofilm, and anti-inflammatory properties. Numerous randomized controlled clinical studies and case reports have been conducted in order to prove its wound-healing efficacy. A meta-analysis investigating the efficacy of honey in different types of wounds indicated that the relative effect of honey on wounds was unclear because the trials performed so far have been small, clinically heterogeneous, short, and highly biased [1,2,3,4,5]. However, some systematic reviews and meta-analyses have indicated that honey dressings are superior in the treatment of diabetic foot ulcers or burns when compared to conventional dressings [6,7,8]. Further clinical trials using honey as a topical wound care product are highly needed to clarify this ambiguity.

An important feature of honey is its antibacterial activity. Honeys exhibiting high antibacterial activity are commercially available as medical-grade honeys. The exact mechanisms of honey antibacterial action may be different depending on the botanical origin of honey. The antibacterial activity of manuka and kanuka honeys are mainly mediated by highly reactive methylglyoxal [9,10,11]. Blossom honeys such as acacia, rapeseed, linden, and sunflower, together with honeydew honeys, exhibit antibacterial activities mainly mediated by the presence of hydrogen peroxide (H_2_O_2_) [12,13]. Indeed, H_2_O_2_ was shown to be a major antibacterial compound of natural honey. H_2_O_2_ is generally produced by glucose oxidase (GOX)-mediated conversion of glucose to gluconic acid under aerobic conditions in diluted honey [14]; however, our recent study suggested that H_2_O_2_ in diluted honeydew honeys is likely also produced via an alternative non-enzymatic pathway, where plant polyphenols may take part in the process of gradual H_2_O_2_ production [12]. In addition, the antibacterial activity of honey can be enhanced through enrichment with antimicrobial peptides [15], α-cyclodextrin [16], or essential oil [17].

Vitamins, including the vitamin B complex and vitamin C (Vit C, ascorbic acid) represent a minor portion of honey. Vit C has been found in almost all types of honey, and its concentration and antioxidant capacity depend on the processing and storage of honey as well as on its botanical origin [18]. A variety of Vit C concentrations have been found in various honey samples, where the concentration of Vit C ranged from 0.34 to 75.9 mg/100 g of honey [19]. Although Vit C is a reducing agent capable of rapidly scavenging reactive oxygen species (ROS), surprisingly, it acts as a pro-oxidant antibacterial molecule [20,21,22] and it may alter the antimicrobial activity of some antibiotics [23,24]. There are two proposed mechanisms of action: (i) the transfer of Vit C into the bacterial cells, resulting in the formation of H_2_O_2_ and, subsequently, ROS and (ii) the generation of lactate and acetic acids from Vit C [23,25]. In addition, the interaction of Vit C with ‘free’, catalytically active metal ions could contribute to oxidative damage through the production of hydroxyl and alkoxyl radicals [20].

Since the combination of natural products is considered a suitable approach to overcome bacterial resistance and inactivate multi-drug resistant bacteria, supplementation of honey with agents such as Vit C may strengthen honey’s antibacterial activity. Therefore, the aim of the present study was to investigate the impact of Vit C on the antibacterial activity of natural honeys against planktonic and biofilm-embedded bacterial pathogens as well as to examine the role of accumulated H_2_O_2_, a major antibacterial compound in honey, in prepared honey mixtures.

## 2. Results

### 2.1. Effect of Vit C on Honey Antibacterial Activity Against Different Bacterial Isolates

**Figure 1:**
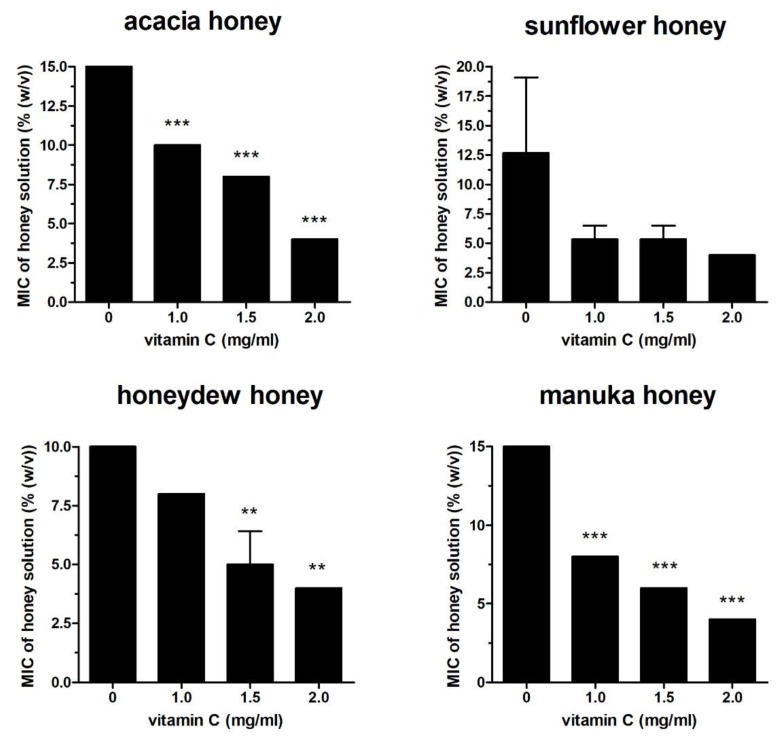
Antibacterial activities of honey samples (acacia, sunflower, honeydew, and manuka honey) supplemented with Vit C at sub-inhibitory concentrations against *Pseudomonas aeruginosa* isolates. The activity was determined with a MIC assay. The MIC was defined as the lowest concentration of honey solution (%) that inhibited bacterial growth. The data are expressed as mean values with standard deviations. ** *P* < 0.01 and *** *P* < 0.001.

**Figure 2:**
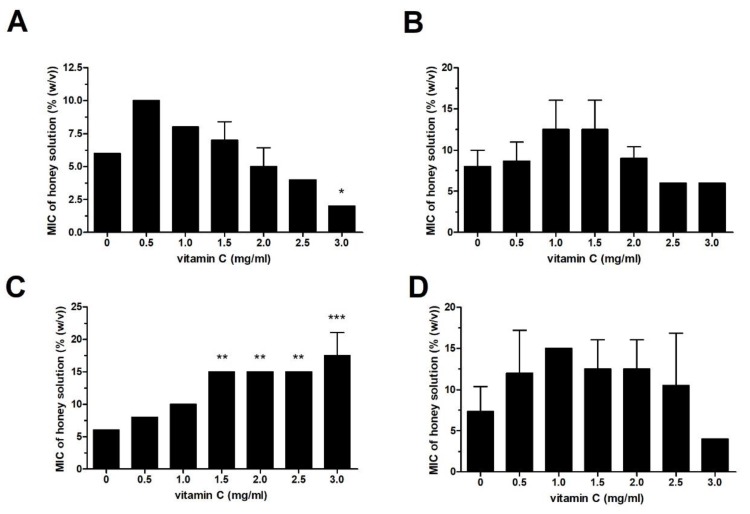
Antibacterial activity of honeydew honey supplemented with Vit C at sub-inhibitory concentrations against bacterial isolates of (**A**) *Escherichia coli*, (**B**) *Serratia marcescens*, (**C**) *Staphylococcus aureus*, and (**D**) *Bacillus subtilis*. The activity was determined with a MIC assay. The MIC was defined as the lowest concentration of honey solution (%) that inhibited bacterial growth. The data are expressed as mean values with standard deviations. * *P* < 0.05, ** *P* < 0.01, and *** *P* < 0.001.

The following four different honey samples were used in the study: acacia, sunflower, honeydew, and manuka. The minimum inhibitory concentration (MIC) values of the honey samples and Vit C against *Pseudomonas aeruginosa* were 10–15% of honey solution and 4 mg/mL, respectively (Figure 1). The antibacterial activities of the honey solutions supplemented with sub-MICs of Vit C (1, 1.5, and 2 mg/mL) increased significantly in a dose-dependent manner. At a sub-MIC of 2 mg/mL, Vit C decreased the MIC values of all honey samples against *P. aeruginosa* to 4% (Figure 1). Honeydew honey, the most effective honey against *P. aeruginosa* with a MIC value of 10%, was used for further testing after its supplementation with sub-MICs of Vit C against a battery of different bacteria (*Serratia marcescens*, *Escherichia coli*, *Staphylococcus aureus*, and *Bacillus subtilis*) (Figure 2A,B). Honeydew honey exhibited even higher inhibitory activity, with MIC values ranging from 6% to 8% depending on the bacterial species, in comparison to its activity against *P. aeruginosa* (Figure 2). On the other hand, supplementation of honeydew honey with sub-MICs of Vit C did not augment the antibacterial potential of the supplemented honey, particularly against Gram-positive bacteria. Furthermore, in the case of *S. aureus*, supplementation with Vit C resulted in an unwanted significant increase in honey MIC values. A significant increase in the antibacterial activity of the supplemented honeydew honey was only achieved against *E. coli* (Figure 2A). The resistance of Gram-positive bacteria to Vit C-supplemented honey may be attributed to differences in the cell wall structure or in cell metabolism.

The fractional inhibitory concentration (FIC) index for the combined application of honey samples and Vit C was 1.0 for *P. aeruginosa*, suggesting an additive interaction of Vit C against this bacterium.

### 2.2. Effect of Vit C on the Antibacterial Activity of Heat- and Catalase-Treated Honey Samples

**Figure 3:**
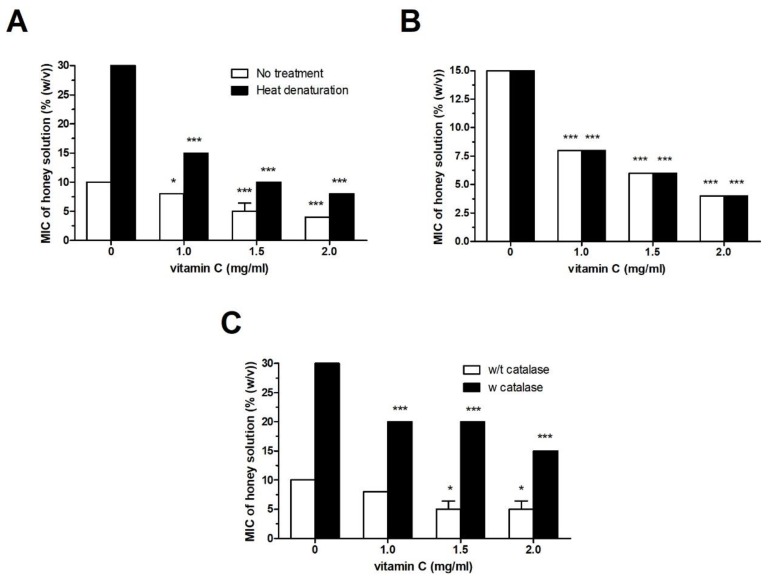
Antibacterial activity of honeydew and manuka honey samples before and after thermal treatment at 100 °C for 5 min against *Pseudomonas aeruginosa* isolates. (**A**) honeydew honey and (**B**) manuka honey. Both honey samples were supplemented with Vit C after thermal treatment. (**C**) Honeydew honey was also subjected to catalase treatment, and the antibacterial activity against *P. aeruginosa* was determined after its supplementation with Vit C at sub-inhibitory concentrations. The activity was determined with a MIC assay. The MIC was defined as the lowest concentration of honey solution (%) that inhibited bacterial growth. The data are expressed as mean values with standard deviations. * *P* < 0.05, and *** *P* < 0.001.

Honeydew and manuka honey samples were heat-treated in order to denature the major compounds responsible for direct (defensin-1) and indirect (GOX and polyphenols) antibacterial activity. Heated honey samples were supplemented with Vit C at final sub-MICs, and the overall antibacterial activity against *P. aeruginosa* was determined. Heat treatment of honeydew honey caused a significant loss of antibacterial activity; however, at sub-MICs, Vit C was able to restore the antibacterial activity of honeydew honey to its original level before heat treatment (Figure 3A). As expected, manuka honey was resistant to heat treatment, and its antibacterial activity with/without Vit C supplementation remained unchanged after exposure to heat (Figure 3B).

Catalase-treated honeydew honey showed weak antibacterial activity due to the neutralization of H_2_O_2_, and this reduced activity was comparable to the heat-affected antibacterial activity. At a concentration of 2 mg/mL, Vit C in the presence of catalase was able to increase the antibacterial activity of honeydew honey, as documented by a change of the MIC value from 30% to 15% (Figure 3C).

### 2.3. Effect of Vit C on H_2_O_2_ Production in Honey Samples

**Figure 4:**
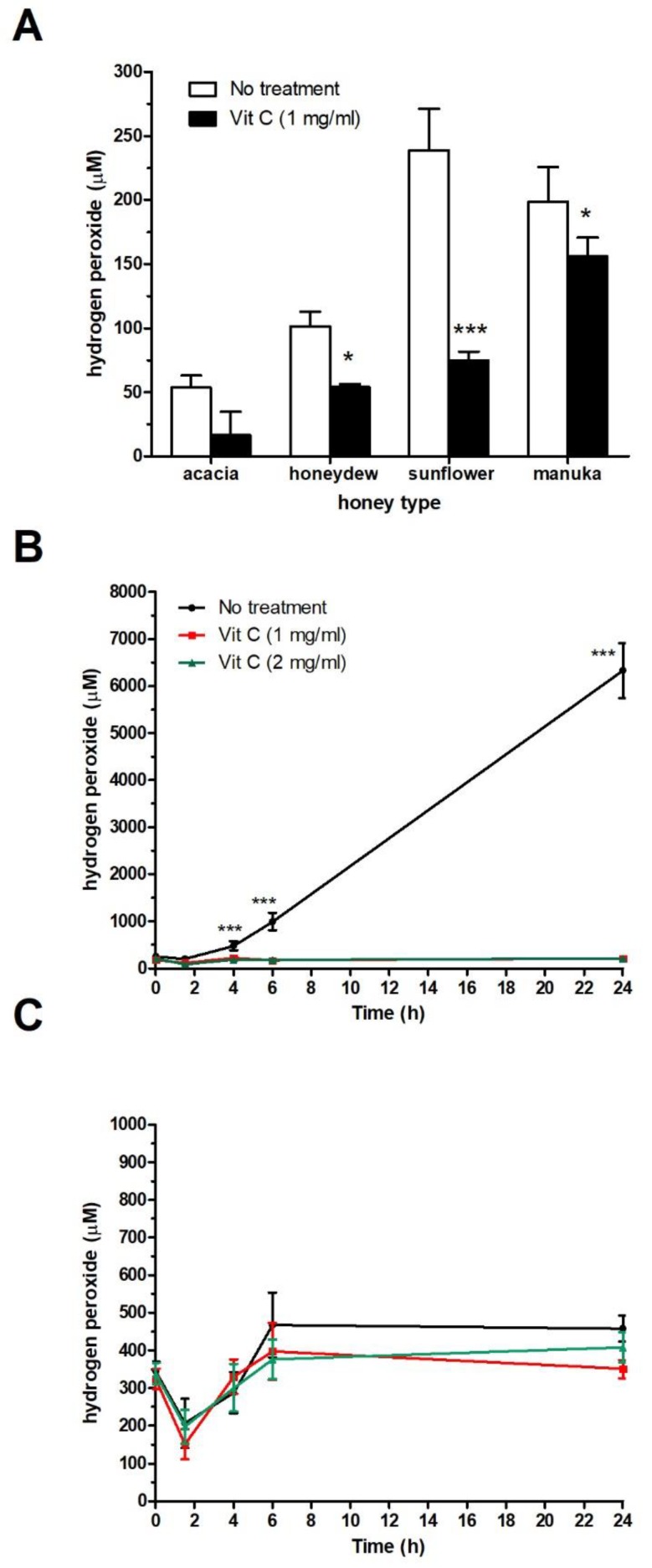
Neutralization of hydrogen peroxide (H_2_O_2_) in different honey solutions after supplementation with vitamin C (Vit C) at sub-inhibitory concentration. (**A**) H_2_O_2_ content was measured in non-incubated, 40% (*w*/*v*) acacia, honeydew, sunflower, and manuka honey solutions supplemented with Vit C with a modified glucose oxidase (GOX) assay kit. (**B**) H_2_O_2_ production was measured in 40% (*w*/*v*) honeydew and (**C**) manuka honey solutions with a modified GOX assay kit after 2, 4, 6, and 24 h of incubation at 37 °C. The data are expressed as mean values with standard deviations. ** *P* < 0.01, and *** *P* < 0.001.

H_2_O_2_ concentration was assessed in freshly prepared 40% honey solutions supplemented with Vit C at a sub-MIC of 1 mg/mL. As shown in Figure 4A, Vit C neutralized H_2_O_2_ in all honey samples, including manuka honey. Furthermore, when the 40% honeydew solution with/without Vit C supplementation at concentrations of 1 and 2 mg/mL was incubated for a certain period of time (up to 24 h) at 37 °C, a significant decrease in H_2_O_2_ production occurred (Figure 4B). Manuka honey, as a non-peroxide honey type, did not generate H_2_O_2_ in the 40% honey solution due to the methylglyoxal-induced loss of GOX activity (Figure 4C).

### 2.4. Effect of Honeydew Honey Supplemented with Vit C Against Bacterial Wound Pathogens Grown in a Multi-Species Biofilm

**Figure 5:**
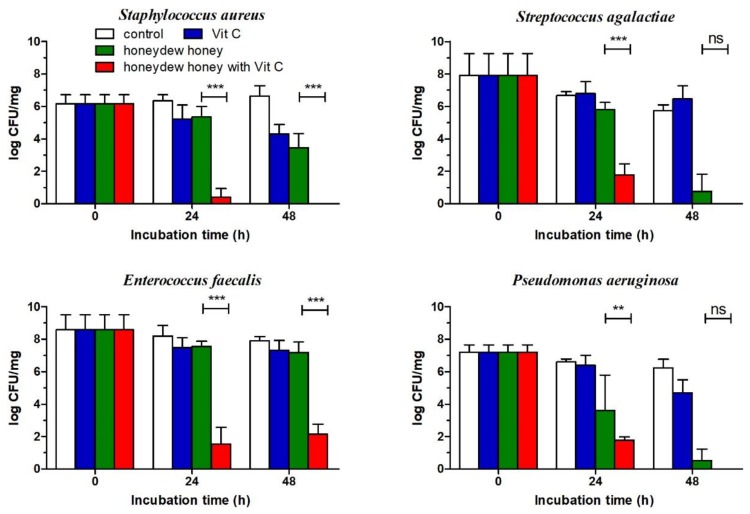
The antibiofilm effects of Vit C (100 mg/mL), honeydew honey, and honeydew honey supplemented with Vit C at a concentration of 100 mg/g of honey against particular bacterial species forming a modified Lubbock chronic wound biofilm after 24 h and 48 h of treatment. The data are expressed as mean values with standard deviations. ** *P* < 0.01; *** *P* < 0.001; ns: non-significant.

Honeydew honey, exhibiting strong antibacterial activity, was used for evaluating honey antibiofilm activity. A multi-species biofilm was formed by four bacterial species (*S. aureus*, *Streptococcus agalactiae*, *P. aeruginosa*, and *Enterococcus faecalis*) and remained stable during the experimental time period. The mature biofilm was treated with honeydew honey at a concentration of 100%, Vit C at a concentration of 100 mg/mL, and a mixture of honeydew honey with Vit C at a concentration of 100 mg/g of honey, and the number of surviving cells was determined by plate counting on different media (Figure 5). Vit C exhibited the weakest antibiofilm activity, and a significant reduction in bacterial viability in the biofilm was documented only for *S. aureus*. Honeydew honey showed strong antibiofilm activity and was able to significantly reduce the cell viability of *S. aureus*, *S. agalactiae*, and *P. aeruginosa* in the mature biofilm within 48 h. On the other hand, in the multi-species biofilm, *E. faecalis* was rather resistant to the antibacterial effect of honeydew honey. The antibiofilm effect of honeydew honey supplemented with Vit C was stronger, and the mixture significantly lowered the counts of all bacterial wound isolates, including *E. faecalis*, within 24 h. Complete eradication of *S. aureus*, *S. agalactiae*, and *P. aeruginosa* in the biofilm was achieved after 48 h of incubation (Figure 5).

## 3. Discussion

One of the important features of honey is its multifactorial antibacterial action, with no risk of development of bacterial resistance. However, the bactericidal efficacy of honey is limited, and some bacterial pathogens could be more resistant to honey than others. In general, honey is more effective against Gram-positive bacteria, except for *E. faecalis*, than Gram-negative bacteria in a planktonic form. In the case of biofilm-embedded bacteria, honey becomes less effective, and a high concentration of a honey solution is needed to eradicate the bacteria within the biofilm. To facilitate improvements in the antibacterial efficacy of honey, particularly against bacterial biofilms, honey has been supplemented with several distinct substances, including antimicrobial peptides [15], bacteriophages [26,27], and essential oils [17]. In the present study, we showed that supplementation of different types of honey with sub-MICs of Vit C resulted in a significant enhancement of the antibacterial activity against *P. aeruginosa* and *E. coli*. On the other hand, Vit C did not improve the antibacterial efficacy of honey against *B. subtilis* or it significantly reduced the antibacterial activity of the tested honey against *S. aureus*. Taking into account the state of the bacteria and the fact that biofilm-embedded bacteria are more resistant to antibacterial compounds, Vit C at a higher concentration (100 mg per g of honey) improved the antibacterial efficacy of honeydew honey against all bacteria including *S. aureus* and *E. faecalis*. Interestingly, the honeydew honey supplemented with Vit C significantly decreased the viability of *E. faecalis*, which was highly resistant to 100% honeydew honey.

*E. faecalis* together with *Enterococcus faecium* demonstrate an intrinsic resistance to common antibiotics and they readily acquire resistance to other novel classes of antibiotics due to a plastic genome [28]. In our previous study [29], within a multispecies biofilm, *E. faecalis* was shown to be resistant to honeydew honey and manuka honey, as well as to the antibacterial peptide defensin-1. These results are in agreement with those of other studies, where *E. faecalis* exhibited the highest resistance among the nosocomial and foodborne pathogens against Corsican and Italian honeys [30,31]. The difficulty of *E. faecalis* treatment has been attributed to the lack of anti-infective strategies to eradicate its biofilm and to the frequent emergence of multidrug-resistant strains. Therefore, effective options are needed to eradicate *E. faecalis*, both in planktonic and in biofilm forms. One potential effective option is a combined treatment where individual agents may act synergistically. Recently, an anti-*E. faecalis* phage combined with vancomycin effectively eliminated an *E. faecalis* biofilm [32].

Vit C, a regular but concentration-variable component of honey, is an important antioxidant agent that significantly decreases the adverse effects of ROS. A surprising observation regarding the pro-oxidant effect of Vit C was reported [20]. Vit C acts as an antimicrobial agent against different bacterial pathogens, including *S. aureus* [23], *E. coli* [21], and *Klebsiella pneumoniae* [22]. It has been proposed that the antibacterial effects of Vit C might be both bacterial strain- and concentration-dependent. The most probable Vit C antibacterial mechanism of action is based on oxidative stress due to the generation of ROS. According to a very recent study [33], the combination of Vit C with an essential oil and copper resulted in free radical generation through the Fenton reaction. Honey, particularly honeydew honey, is rich in minerals including transition metal ions (Fe and Cu) and polyphenols, and its combination with Vit C may trigger the intracellular production of ROS in bacterial cells. At sub-MICs, Vit C in a honey solution rapidly neutralizes the generated H_2_O_2_, which is a major antibacterial factor of honey. The antibacterial activity of the honey/Vit C combination against certain Gram-positive bacteria in planktonic cultures is less effective or ineffective, which can be attributed to differences in the bacterial cell wall structure, ROS-protective enzymes, or metabolism.

Discrepancies in the antibacterial efficacy of honey supplemented with Vit C against planktonic and biofilm cultures of *S. aureus* were reported in this study. There could be several reasons for these discrepancies, but the most important is the fact that a multi-bacterial wound biofilm consisting of four different bacterial isolates, including *S. aureus*, was utilized. From a clinical point of view, honey supplemented with Vit C mediated the eradication of all species in the multispecies biofilm, which was achieved after 24 h. Therefore, the honey/Vit C combination represents a very promising therapy for the treatment of chronically infected wounds, which needs to be evaluated clinically.

A combination of honey and Vit C has already been pre-clinically, topically tested in an animal, non-infected, burn wound model [34]. It was shown that the combination of honey with Vit C promoted rapid debridement, and an advanced proliferative phase was observed with respect to unsupplemented honey. Unfortunately, no clinical data are currently available for the topical application of the combined therapy (honey and Vit C) in wound care management. Further clinical studies are warranted in order to determine the potential therapeutic effect of Vit C-supplemented honey.

## 4. Materials and Methods

### 4.1. Honey Samples

The following honey sample types were collected across Slovakia and used in this study: acacia, sunflower, linden, and honeydew. Commercially available manuka honey with a unique manuka factor of 15 (UMF 15+), imported from New Zealand, was purchased from Nature’s Nectar (Surrey, UK).

### 4.2. Bacterial Strains for Antibacterial and Antibiofilm Activity Testing

The antibacterial activities of honey samples, Vit C, as well as a mixture of honey and Vit C were assessed against *P. aeruginosa* CCM1960 and *S. aureus* CCM4223 isolates, obtained from the Department of Medical Microbiology, Slovak Medical University (Bratislava, Slovakia), as well as against *E. coli* CCM3954, *S. marcescencs* CCM8587, and *B. subtilis* CCM2216 isolates obtained from the Institute of Chemistry Slovak Academy of Sciences (Bratislava, Slovakia).

Bacterial strains of *S. aureus*, *S. agalactiae*, *E. faecalis*, and *P. aeruginosa* which were originally isolated from patients with chronic wounds hospitalized at University Hospital, Hradec Kralove (Hradec Kralove, Czech Republic), were used for multi-species wound biofilms (modified Lubbock chronic wound biofilm model). Sodium chloride peptone broth (buffered peptone bouillon, BPB; Merck, Darmstadt, Germany) was used for dilutions and for normalizing the optical density of the cultures in all wound biofilm experiments.

### 4.3. Determination of the Antibacterial Activities of Honey, Vitamin C (Vit C), and a Mixture of Honey and Vit C

The antibacterial efficacies of the honey samples were evaluated with a MIC assay, as described by Bucekova et al. (2019) [13]. First, an overnight bacterial culture was suspended in phosphate-buffered saline (PBS) (pH 7.2), and the turbidity of the suspension was adjusted to 10^8^ colony-forming units (CFU) per milliliter and diluted with Muller–Hinton medium (MHB) to a final concentration of 10^6^ CFU/mL. Then, 10 μL aliquots of the suspension were inoculated into each well of sterile, 96-well, polystyrene, U-shape plates (Sarstedt, Nümbrecht, Germany). The final volume in each well was 100 μL, consisting of 80 μL of diluted honey (with MHB), 10 μL of a Vit C (Sigma-Aldrich, Taufkirchen, Germany) solution at different concentrations (0.5, 1, 1.5, 2, 2.5, and 3 mg/mL), and 10 μL of the bacterial suspension. Serial dilutions of each honey sample were prepared from a 50% (*w*/*w*) honey solution, resulting in final concentrations of 40%, 35%, 30%, 25%, 20%, 18%, 16%, 14%, 12%, 10%, 8%, 6%, 4%, and 2%.

After 18 h of incubation at 37 °C, bacterial growth inhibition was determined by monitoring the optical density at 490 nm. The MIC was defined as the lowest concentration of honey, Vit C, and mixture of honey with Vit C that inhibited bacterial growth. All tests were performed in triplicate and repeated three times.

The FIC index was calculated according to the following equation: FIC index = FIC (honey) + FIC (Vit C) = (MIC of honey in combination/MIC of honey alone) + (MIC of Vit C in combination/MIC of Vit C alone). The interaction was defined as synergistic if the FIC index was ≤ 0.5, additive if the FIC index was ≥ 0.5 and ≤ 1.0, and indifferent if the FIC index was > 1.0 and ≤ 2.0 [35].

### 4.4. Antibiofilm Activity Analysis Using a Polybacterial Biofilm

We used the previously described modified Lubbock chronic wound biofilm model according to Sojka et al. (2016) [29]. First, 6 mL of medium, containing Bolton broth base (Sigma-Aldrich, Germany), 1% gelatin, 50% porcine plasma, and 5% porcine erythrocytes lysed by freeze–thawing, was dispensed into sterile 1.6 × 10 cm glass tubes. Cultures of the selected bacteria, normalized according to the optical density, were mixed, and 10 µL of the mixture containing 10^6^ CFU/mL was inoculated into each tube by ejecting the pipette tips along with the bacterial suspension. Inoculated tubes were incubated at 37 °C in an orbital shaker (1500 g) for 48 h, and the pre-formed biofilms were harvested.

Harvested biofilms were washed with BPB. The biofilms were placed into the artificial wound bed in the nutrient medium and covered with a piece of 100% cotton 8-ply gauze sponge (Batist medical, Cerveny Kostolec, Czech Republic), soaked with the test substance (100% honeydew honey or its mixture with Vit C—100 mg of Vit C per g of honey) or BPB (negative control). Biofilms in the artificial wound bed treated with particular substances were incubated at 37 °C for 24 h and 48 h. After treatment, the biofilms were harvested from the artificial wound bed using sterile forceps and a Lang eye spoon and then homogenized. The bacteria per culture were determined using different media, as described before [36], and Brilliance UTI selective medium (Oxoid, Basingstoke, UK).

### 4.5. Determination of Hydrogen Peroxide H_2_O_2_ Concentration

The concentration of H_2_O_2_ in the honey samples was determined with the Megazyme GOX assay kit (Megazyme International Ireland Ltd.), which is based on the release of H_2_O_2_. For the standard, H_2_O_2_ diluted to 9.8–312.5 μM was used. Briefly, 40% (*w*/*w*) honey solutions in 0.1 M potassium phosphate buffer (pH 7.0) were prepared and either immediately used for H_2_O_2_ measurements or measured after incubation of the prepared solutions for 24 h at 37 °C. Each honey sample and standard were tested in duplicate in a 96-well microplate, and the absorbance was measured at 510 nm using a Synergy HT microplate reader (BioTek Instruments, Winooski, VT, USA).

### 4.6. Heat and Catalase Treatment of Honey

Samples of 100% honeydew honey and manuka honey were heated at 100 °C for 5 min in order to destroy the antibacterial activity mediated by GOX, polyphenols, and defensin-1. The samples were then cooled to room temperature and used alone or supplemented with Vit C at sub-MICs for the determination of the antibacterial activity against *P. aeruginosa*.

A diluted honeydew honey sample (50% *w*/*w* in MHB medium) was treated with catalase (2000–5000 U/mg protein; Sigma-Aldrich) at a final concentration of 1000–2500 U/mL at room temperature for 2 h in order to eliminate H_2_O_2_, the major antibacterial factor of honey. Catalase-treated honeydew honey was then used alone or supplemented with Vit C at sub-MICs in the antibacterial assay to determine MIC values against *P. aeruginosa*.

### 4.7. Statistical Analysis

All data were statistically analyzed using one-way ANOVA. The data are expressed as mean values with standard deviations (SDs). Data with *P* values smaller than 0.05 were considered statistically significant. All statistical analyses were performed using GraphPad Prism (GraphPad Software Inc., La Jolla, CA, USA).

## 5. Conclusions

In conclusion, our present study shows that supplementation of honey with Vit C at sub-MICs significantly enhances the antibacterial activity of honey against *P. aeruginosa* and *E. coli* planktonic cultures. On the other hand, the combined therapy was shown to be ineffective against *S. aureus* planktonic cultures. However, when a 100% honey supplemented with Vit C at concentration of 100 mg/g of honey was used in a multispecies wound biofilm model, complete eradication of all bacterial isolates, including *S. aureus*, was observed. Furthermore, the combined therapy was effective against *E. faecalis*, whereas honey alone exhibited no antibacterial activity against this bacterium. The exact mechanism of action of Vit C-supplemented honey is unclear; however, honey polyphenols and mineral contents in the presence of Vit C could be responsible for intracellular free radical generation in bacterial cell.

## 6. Patents

Some of the data in this publication have been used in the national utility model application 8435 and are owned by the Institute of Molecular Biology Slovak Academy of Sciences and Slovak Medical University.
